# PESTICIDES: Toward DDT-Free Malaria Control

**DOI:** 10.1289/ehp.117-a344

**Published:** 2009-08

**Authors:** Adrian Burton

**Affiliations:** **Adrian Burton** is a biologist living in Spain who also writes regularly for *The Lancet Oncology, The Lancet Neurology, and Frontiers in Ecology and the Environment.*

An increased international effort to reduce the incidence of malaria around the globe while reducing reliance on DDT was announced 6 May 2009 at the fourth meeting of the Conference to the Parties to the Stockholm Convention on Persistent Organic Pollutants (POPs). With funding of more than US$70 million, the United Nations Environment Programme and the World Health Organization have launched 10 projects to help test integrated vector management (IVM) systems for malaria control. These systems could provide sustainable, effective, and cost-effective alternatives to reliance on DDT. The aim is to reduce DDT application by 30% over current usage by 2014, with a complete phaseout by the early 2020s. About half the funding for the projects comes from the Global Environment Facility (GEF), the financial arm of the Stockholm Convention, which provides financial and technical assistance to help countries phase out and reduce releases of POPs.

DDT is banned for all uses in all countries that are signatories to the Stockholm Convention except for spraying inside buildings in developing countries where malaria is a problem. This practice, known as indoor residual spraying (IRS), is increasingly relied upon in Africa and Asia, given the resurgence in malaria in recent decades. Reports also exist that, on occasions, some developing countries contravene the Stockholm Convention and on a larger scale. growing evidence resistance to DDT as well as adverse human health effects has prompted a search for alternatives.

“There is a large and growing body of literature on the potential human health effects of DDT,” says Brenda Eskenazi, a professor of epidemiology and maternal and child health at the University of California, Berkeley. “Evidence suggests that exposure to DDT and its breakdown product DDE at levels substantially lower than that experienced in communities that use IRS may be associated with breast cancer, diabetes, spontaneous abortions, decreased semen quality, and impaired child neurodevelopment.”

Great care is taken and safeguards followed in the new projects—which involve 40 countries across Africa, the Eastern Mediterranean, and Central Asia—to ensure that malaria incidence does not increase in the project areas, says Laurent Granier, coordinator of the Chemicals Cluster for the GEF. The new projects follow a successful pilot project in Mexico and Central America that achieved an overall 63% reduction in the incidence of malaria and a more than 86% reduction in the most severe form of malaria, that caused by *Plasmodium falciparum*. This success has rekindled hopes that an end to DDT reliance is possible.

IVM achieves such reductions with strategies such as using insecticide-treated bed nets, draining standing water from ditches, adding fish that feed on mosquito larvae to water supplies, clearing vegetation cover for adult insects, and improving diagnostic and treatment capacity to reduce the reservoir of blood parasites in the human population. According to the Centers for Disease Control and Prevention, the only insecticides approved for use on bed nets are pyrethroids, which are less acutely toxic to mammals than other pesticides.

From a public health viewpoint, IVM needs to match DDT in terms of controlling malaria. But other comparisons are more difficult, due in part to a lack of cost-effectiveness data from communities that currently use IRS. “Comprehensive cost-effectiveness analysis is very laborious, requiring large-scale trials to accurately determine the effect on disease in targeted populations,” explains Henk van den Berg, an entomologist and visiting scientist at Wageningen University. “Moreover, the effects measured in one place may not have wide-scale application value because they are influenced by local variables. Also, costs should not be limited to program costs but include indirect costs due to side effects.”

One way to tackle this complex issue is to study how each intervention affects vector and disease transmission in different locations, says van den Berg. In a project being undertaken in Kenya, Tanzania, and Uganda, researchers from Duke University are developing the Malaria Decision Analysis Support Tool (MDAST), which applies existing knowledge and evidence about specific malaria interventions to help officials predict the probable outcomes of different combinations of malaria control strategies and weigh the risks and benefits. They can then make the best choices for disease management and vector control given the environmental and societal parameters of specific situations.

“Controlling malaria requires a coordinated adaptive decision-making approach based on the best available evidence from the field,” explains MDAST developer Randall Kramer, a professor of resource and environmental economics at Duke. Kramer and colleagues described the MDAST in an article published 6 April 2009 ahead of print in *Health Policy*.

A potential problem faced by IVM is the lack of sustained interest by communities that are responsible for the maintenance of IVM strategies—for instance, ensuring that water does not re-collect in ditches. But properly implemented IRS strategies also require sustained input as well as an expensive-to-maintain infrastructure. Hopefully, the results of these projects will allow developing countries to reduce their dependence on DDT, thus avoiding its potentially harmful effects on human health and the environment, while reducing the prevalence of malaria—a disease that has killed five children in the time it took you to read this article.

## Figures and Tables

**Figure:**
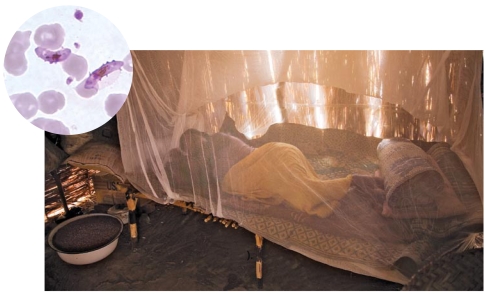
Kerfi, eastern Chad (inset: *P. falciparum*). Treated bed nets are one potentially safer alternative to DDT in fighting malaria.

